# Universal Features in Panarthropod Inter-Limb Coordination during Forward Walking

**DOI:** 10.1093/icb/icab097

**Published:** 2021-06-28

**Authors:** Jasmine A Nirody

**Affiliations:** Center for Studies in Physics and Biology, Rockefeller University, New York, NY 10065, USA; All Souls College, University of Oxford, Oxford, OX1 4AL, UK

## Abstract

Terrestrial animals must often negotiate heterogeneous, varying environments. Accordingly, their locomotive strategies must adapt to a wide range of terrain, as well as to a range of speeds to accomplish different behavioral goals. Studies in *Drosophila* have found that inter-leg coordination patterns (ICPs) vary smoothly with walking speed, rather than switching between distinct gaits as in vertebrates (e.g., horses transitioning between trotting and galloping). Such a continuum of stepping patterns implies that separate neural controllers are not necessary for each observed ICP. Furthermore, the spectrum of *Drosophila* stepping patterns includes all canonical coordination patterns observed during forward walking in insects. This raises the exciting possibility that the controller in *Drosophila* is common to all insects, and perhaps more generally to panarthropod walkers. Here, we survey and collate data on leg kinematics and inter-leg coordination relationships during forward walking in a range of arthropod species, as well as include data from a recent behavioral investigation into the tardigrade *Hypsibius exemplaris*. Using this comparative dataset, we point to several functional and morphological features that are shared among panarthropods. The goal of the framework presented in this review is to emphasize the importance of comparative functional and morphological analyses in understanding the origins and diversification of walking in Panarthropoda.

Introduction

Walking, a behavior fundamental to numerous tasks important for an organism’s survival, is assumed to have become highly optimized during evolution. Terrestrial animals must navigate rough, varying landscapes; as such, stepping patterns must be flexible to successfully complete a range of behavioral goals across a range of terrains. The foremost of these adaptations is variability in the temporal and spatial coordination between leg movements. In vertebrates, this variability manifests as distinct gaits: for example, a horse will switch from a walk to a trot to a gallop as it increases forward speed (Fig. 1). These switches are generally driven by energy optimization processes and accompanied by changes in the movements of the animal’s center of mass (COM) as well as discontinuities or sharp transitions in at least one parameter, e.g., duty factor or the phase offset between leg pairs (Alexander 1989; Alexander and Jayes 2009).

**Fig. 1.**
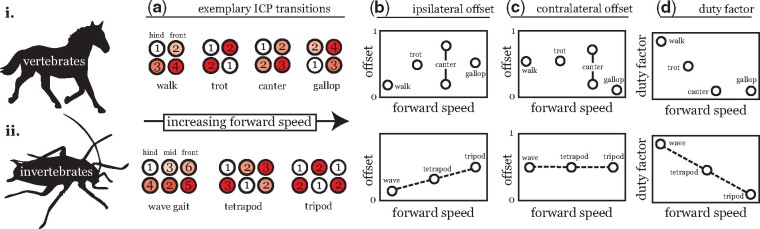
Transitions in stepping pattern with walking speed in vertebrates (**i**) and invertebrates (**ii**). Both vertebrates and invertebrates show changes in ICPs with speed. While our discussion focuses on leg kinematics, it is important to note that gait transitions comprise both changes in leg coordination and body dynamics, as has been extensively documented in vertebrate species (Alexander 1989). Representative ICP transitions for (i) tetrapods and (ii) hexapods are shown. (**A**) Horses transition from a walk at low speeds, to a trot at intermediate speeds, to a canter or gallop at high speeds, while insects switch from pentapodal wave to tetrapodal and then tripod coordination as walking speed increases. Numbering denotes the order of footfalls within a full stride cycle; the timing of footfalls is also denoted from lighter to darker coloring. Stepping patterns in vertebrates can be categorized into discrete gaits that are mirrored by transitions in body dynamics driven by energy optimization processes. These transitions show discontinuities in parameters such as phase offset between leg pairs (**B and C**) and duty factor (**D**). Note that, as a three-beat gait, the canter is asymmetric and exhibits different characteristic phase offset between the two ipsilateral and contralateral leg pairs. In contrast, invertebrate walking is a continuum with intermediate stepping patterns providing smooth transitions between “canonical” stepping patterns. although the “canonical” stepping patterns shown here correspond to hexapods, observed trends for phase offsets and duty factor are generalizable to arthropods with any number of legs: (**B**) ipsilateral phase offset increases continuously with walking speed, (**C**) contralateral phase offset is anti-phase across speeds, and (**D**) duty factor decreases continuously with walking speed (Manton 1952a).

At first glance, similar transitions with walking speeds are present in arthropod species. Slow walking insects largely use a wave coordination, in which at most one leg is lifted (in the “swing” phase) at a time. Insects walking at intermediate speeds utilize tetrapodal stepping patterns, in which two limbs enter the swing phase simultaneously. Finally, fast-running insects employ tripod coordination, in which two pairs of three legs each lift-off in sequence; each tripod comprises an ipsilateral front and hind leg and the contralateral middle leg. A schematic illustrating these canonical patterns is shown in Fig. 1. While inter-leg coordination patterns (ICPs) in insects are often referred to as “gaits” in the literature (Nishii 2000; Dürr et al. 2004; Bender et al. 2011), it has yet to be explicitly shown that transitions between invertebrate ICPs with speed constitute transitions between discrete gaits (Alexander 1989).

The vast majority of recent studies on arthropod locomotion consist of deep investigation into the behavior of a single organism (most commonly, an insect). Within this framework, our understanding of the nature of *transitions* between invertebrate ICPs is hindered by the fact that most species use a limited range of spontaneous walking speeds under constrained laboratory conditions (e.g., forward walking on flat, uniform terrain). Such controlled trials do not allow for the observation of switches between preferred stepping patterns. Ants, for example, have been recorded using primarily tripod coordination across a speed range of approximately 5–30 body lengths/s (Reinhardt and Blickhan 2014; Wahl et al. 2015; Pfeffer et al. 2019); little data are available at lower speeds that may call for different preferred stepping patterns. Adult stick insects, on the other hand, scarcely walk on flat surfaces at speeds above 1 body length/s and thus rarely have been observed displaying tripodal coordination patterns in the laboratory (Graham 1972; Cruse 1990).

Accordingly, this framework has led to the development of several models of walking, each derived from the behavior of a single, highly specialized organismal system (Ayali et al. 2015). For example, behavioral studies conducted in slow-walking stick insects have suggested that a small set of local coordination rules suffices to explain observed ICPs (Cruse 1990; Dürr et al. 2004). Inter-segmental neural pathways have also been shown to be important in coordinating leg movements in fast tripodal walkers like the cockroach *Periplanta americana* (Pearson and Iles 1973), but a clear connection between these postulated mechanisms has not been rigorously characterized.

Studies on species that exhibit a wide range of preferred walking speeds in the laboratory have been useful in connecting the mechanisms underlying slow and fast walking. One such organism is the fruit fly *Drosophila*, a species for which tool availability is an added benefit: *Drosophila’*s status as a model organism allows for the collection of large datasets and tractable genetic manipulation of neural signals (Mendes et al. 2013; Wosnitza et al. 2013; Szczecinski et al. 2018). These studies have shown that *Drosophila* show ICPs that fall along a speed-dependent continuum containing all the “canonical” stepping patterns observed in other insects (DeAngelis et al. 2019).

Excitingly, these findings have strong implications for our understanding of the underlying locomotor control circuits and corroborate theoretical investigations suggesting that the same circuit may be able to generate the entire observed range of ICPs in *Drosophila* (that is, there are not separate dedicated controllers for, e.g., tripod coordination) (Wosnitza et al. 2013; Schilling and Cruse 2020). Furthermore, the stepping patterns characterized in slow- and fast-walking *Drosophila* closely matched those in both stick insects and cockroaches, respectively (DeAngelis et al. 2019; Schilling and Cruse 2020). Importantly, this leads to the hypothesis that the underlying control circuit responsible for generating the spectrum of ICPs observed in *Drosophila* may be common to all insects, and perhaps more generally to all panarthropods. This hypothesis is consistent with early observations that stepping patterns in Onychophora (velvet worms, which along with Tardigrada and Arthropoda, comprise Panarthropoda) are “sufficiently wide to provide a common origin for all the more specialized types of arthropodan gait” (Manton 1952a).

A simple model put forward based on behavioral analyses in *Drosophila* suggests that walking involves connections between the neuropil of the ventral nerve cord (VNC) (DeAngelis et al. 2019). The arthropod central nervous system shares a common blueprint, consisting of a brain and a series of segmented bilateral ganglia from which lateral nerves extend into each body segment and appendages (Niven et al. 2008; Carmen Ramona Smarandache-Wellmann 2016; Yang et al. 2016). This topology is largely conserved throughout Arthropoda, although it is important to note that there exists significant diversity in ganglionic structure among arthropod classes within this general framework. For example, crustacean ganglia are not completely fused at the midline and display a ladder-like structure, in which hemiganglia are connected by axons within each segment (Storch and Welsch 2014). To this end, integrative studies that consider both functional and phylogenetic relationships among various organismal systems are vital to our understanding of invertebrate walking (Ayali et al. 2015).

In this review, we gather kinematic data on arthropod forward walking on flat surfaces. We note that our analysis is limited by data availability in the literature in two ways. First, the majority of our discussion emphasizes walking kinematics in insects, which is simply a reflection of the distribution of past research in the field; in particular, recent work emphasizing the collection of large kinematic and behavioral datasets has focused almost exclusively on insect species. We attempt to include examples (and data) from a diversity of noninsect arthropods whenever possible. We present these alongside results from our investigations in the eutardigrade *Hypsibius exemplaris* (Nirody et al. 2021). We root our comparisons in a review of nervous system diversification across Panarthropoda (Niven et al. 2008; Carmen Ramona Smarandache-Wellmann 2016) noting, in particular, the similarities in VNC topology between tardigrades and arthopods (Yang et al. 2016). We further describe several exceptions (e.g., “galloping” in some beetles (Smolka et al. 2013)) that diverge from the “canonical” patterns as systems of interest for developing insight into possible adaptive mechanisms for performance in challenging environments.

Second, we constrain our discussion to ICPs, rather than “gaits”; it is important to note that true gaits cannot be defined simply by leg kinematics but must also take the animal’s inertia into account. Gait transitions are driven by energy optimization processes and must be accompanied by changes in body dynamics (Hoyt and Taylor 1981). Recent studies suggest that transition between ICPs in invertebrates may similarly be driven by an optimization against physical constraints (Nishii 2000; Szczecinski et al. 2018). However, data concerning changes in COM dynamics in the literature are available only for a limited number of arthropod species (.g., Full and Tu 1990, 1991; Ting et al. 1994; Dallmann et al. 2017). Given this, our analysis centers on inter-limb coordination, for which large datasets are more readily available (Mendes et al. 2013; Wosnitza et al. 2013; DeAngelis et al. 2019). This focus is encouraged by recent work suggesting that, in addition to mechanical considerations, animals with small circuits for controlling limbs may prefer particular stepping patterns that rely on simple underlying control (DeAngelis et al. 2019). With this work, we hope to highlight the value of performing comparative functional and morphological studies—and, accordingly, the importance of making organismal data open and accessible—in illuminating the origins and evolution of invertebrate walking patterns.

## Methods

**Fig. 2.**
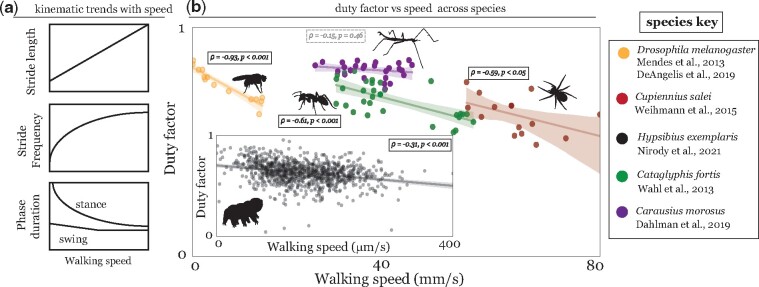
Relationship between kinematic parameters and forward walking speed across panarthropods. (**A**) Schematic depicting generalized relationships between walking speed and stride length (top), stride frequency (middle), and relative stance/swing duration (bottom). Both stride length and frequency increase with walking speed, with stride frequency plateauing at high speeds (Manton 1952a). Each step is composed of a swing (leg lifted) and stance (leg on the ground) period. Speed is largely modulated by changes in stance duration; in contrast, swing duration remains relatively constant over walking speeds, decreasing slowly with walking speed at low to medium speeds and leveling off at high speeds. Recent work has shown little correlation between swing duration and walking speed (Wosnitza et al. 2013; Nirody et al. 2021) (**B**) The relative modulation of swing and stance duration within a stride is characterized by the duty factor, or proportion of a stride spent in stance. All organisms surveyed display a smoothly decreasing duty factor with forward walking speed. Apart from the broadly observed negative correlation across species, the reported relationship between walking speed and duty factor varies in the literature (e.g., Wahl et al. 2015; Weihmann et al. 2017; Pfeffer et al. 2019; Nirody et al. 2021). In the absence of a generalizable underlying model (A constant swing duration and hyperbolic stance duration suggests a hyperbolic relationship between walking speed and duty factor. However, several variations between species (e.g., in the relationship between swing duration and walking speed) result in a range of reported duty factor vs speed relationships, from linear (Pfeffer et al. 2019), to hyperbolic (Wahl et al. 2015), to more complex nonlinear relationships (Weihmann et al. 2017).), we have provided linear fits to guide the eye. We note that walking speed along the *x*-axis is not normalized using body length; this choice of metric is simply a reflection of data availability in the literature. As such, tardigrade data are shown in the inset due to size differences from the other organisms represented.

Data for arthropod species in Figs. 2 and 4 were extracted from published articles as cited. For some articles, tabular data were not available; in these cases, data were extracted from paper figures using the R package *digitize* (Poisot 2011). For inter-leg phase offsets, only mean values are shown for all species other than *Drosophila*, due to the large variation in data availability across studies.

For studies in which distributions of phase offsets between leg pairings were reported, distributions were tested against the normal distribution using the Kolmogorov–Smirnov test at the 5% significance level. Mean values for ipsilateral phase relationships are reported only if phase offsets were normally distributed. This is because a joint distribution of inter-leg phase offset and walking speed was rarely available, and so we attempt to avoid averaging offsets over a large range of walking speeds (e.g., pooling data from a tripod and tetrapod coordination patterns). All available contralateral phase distributions showed a single peak and were normally distributed.

All data shown for the tardigrade *H. exemplaris* were collected as reported in our previous work (Nirody et al. 2021). These data will be made available at http://www.github.com/jnirody/waterbears; all other digitized data shown will be available at http://www.github.com/jnirody/invertICPs.

## Results and discussion

### Invertebrate kinematics vary smoothly with walking speed

Organisms walk to complete a variety of behavioral goals and must be able to do so successfully in a variety of natural environments. To this end, virtually all legged animals have developed strategies to modulate several performance metrics, including, importantly, walking speed (Heglund et al. 1974; Heglund and Taylor 1988). While some kinematic trends with walking speed are generalizable across invertebrate species (Fig. 2a), there are several distinct differences in how different species utilize the interplay between the tuning of various temporal and spatial parameters.

Two intuitive candidates for such parameters are *stride length* and *stride frequency*. Like quadrupeds and bipeds (including humans), invertebrates tune both the length of their steps and the amount of time devoted to each step to modulate their speed of locomotion. Stride length generally shows a linear relationship with speed across walking speeds (Wosnitza et al. 2013; Weihmann et al. 2017; Szczecinski et al. 2018; Clifton et al. 2020). The maximum stride length achievable by an organism is dictated by absolute leg length, unless stride length can be further increased by inserting aerial phases into the stepping pattern. While aerial phases are commonly observed in vertebrate species (e.g., in horse trots or human running), fast-running insects almost always maintain a grounded alternating tripod pattern over a wide range of speeds (Full and Tu 1991; Goldman et al. 2006; Wosnitza et al. 2013; Reinhardt and Blickhan 2014; Wahl et al. 2015; Dallmann et al. 2019; Pfeffer et al. 2019). Only rare instances of aerial phases in high-speed running have been observed in certain individuals (cockroach, *Periplanta americana*: (Full and Tu 1991); ant, *Cataglyphis fortis* (Wahl et al. 2015); spiders, *Hololena adnexa* and *Hololena curta* (Spagna et al. 2011)). Arthropods with higher leg numbers (e.g., arachnids, myriapods) can reach even greater speeds than hexapods during grounded running (Manton 1952b; Spagna et al. 2011).

To increase stride frequency, organisms can reduce the step cycle period by either shortening the *swing* or *stance* phase of the cycle. Each leg’s stride comprises a protraction (swing), in which the leg is lifted and takes a step, and a retraction (stance), in which the leg is in contact with the ground and generates propulsion. Walking speeds across panarthropod species are mainly modulated by stance duration. In contrast, swing duration generally decreases only slightly with speed at low to medium speeds and is constant at high speeds (Fig. 2a; see also, e.g., (Mendes et al. 2013; Wosnitza et al. 2013; Dürr et al. 2018)). This observed trend has lent support to the idea that mechanically mediated load-based coordination is a widespread control strategy (Szczecinski et al. 2018).

This relative modulation is cleanly characterized by changes in the *duty factor—*the proportion of a cycle spent in the stance phase. Transitions between discrete ICPs are often characterized by sudden changes in the duty factor: for example, the walk–trot transition in horses is accompanied by a sharp drop in the animal’s duty ratio from approximately 0.6 to 0.5 (Hoyt et al. 2006, Starke et al. 2009). In line with the hypothesis that insect walking lies along a speed-dependent continuum, all panarthropods surveyed (including several insect species, crustaceans, spiders, and tardigrades) during forward walking on flat surfaces show a smooth, continuous relationship between duty factor and walking speed (Fig. 2b). Arthropods with a large number of legs display significantly lower duty factors than hexapods can achieve at the highest walking speeds; for example, some species of myriapods have been observed to run with only 3 out of 40 legs in the stance phase, corresponding to a duty factor of 0.075 (data not shown; see (Manton 1952b, 1954, 1972) for further details).

### Swing–stance relationships generate smooth transitions

Changes in locomotor output are not limited to tuning the movements of single legs but also include shifts in the temporal coordination among legs. Inter-leg coordination parameters are thought to be of secondary importance with respect to modulation of walking speed but are essential for static and dynamic stability (Szczecinski et al. 2018). Although the literature often refers to “gaits” in insects, there is little evidence that invertebrates show discontinuous transitions in kinematics across forward walking speeds (Fig. 2).

**Fig. 3.**
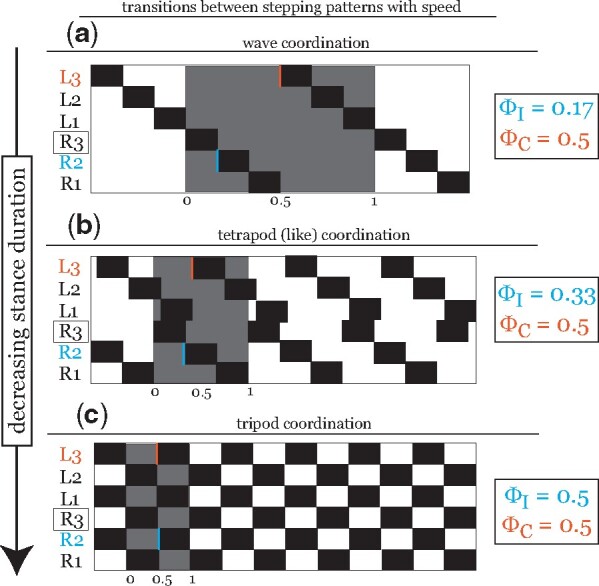
Observed hexapod stepping patterns form a speed-dependent continuum of ICPs generated by modulating a single parameter, stance duration. During forward walking, arthropods transition through a spectrum of ICPs with walking speeds by modulating a single parameter: the duration of stance (duration of ground contact time) (DeAngelis et al. 2019). The duration of swing (duration of a cycle for which a leg is lifted) is kept constant across stepping patterns (Wosnitza et al. 2013; Nirody et al. 2021). Footfall diagrams show a temporal sequence of ground contacts for these three observed patterns. A full cycle (0,1) with respect to reference leg R3 is shown highlighted in grey. Swing is shown in black; stance is shown in white. The relative phase offset of swing initiations by the ipsilateral anterior leg (R2, blue) and the contralateral leg (L3, orange) are denoted for each ICP. Each ICP is defined by a characteristic set of phase offsets between ipsilateral ($\phi_{I}$, blue) and contralateral ($\phi_{C}$, orange) leg pairs. Ipsilateral phase offsets increase with forward walking speed, saturating in most arthropod species at $\phi_{I}=0.5$; contralateral phase offset $\phi_{C}=0.5$ remains constant across walking speeds. In hexapods, three “canonical” stepping patterns along this spectrum have been characterized: (**A**) wave coordination at slow speeds to (**B**) tetrapodal coordination at intermediate speeds to (**C**) tripod coordination at high speeds. Note that a “canonical” tetrapod pattern comprises a sequence of simultaneous lift-offs by three sets of two legs. This results in a contralateral offset of $\phi_{C}=\frac{1}{3}$ (or $\phi_{C}=\frac{2}{3}$ for the mirror-image tetrapod). However, a cross-body offset in step timing such that limbs that are meant to swing simultaneously are actually slightly offset in time results in a tetrapod-like stepping pattern that shows the anti-phase contralateral phase relationship consistent with the observed continuum (DeAngelis et al. 2019).

**Fig. 4.**
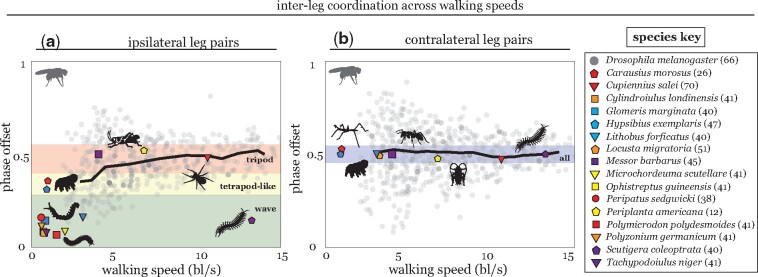
Changes in inter-leg coordination with walking speed. Relationship between walking speed and measured phase offset in swing initiations between (**A**) ipsilateral and (**B**) contralateral leg pairs. Ipsilateral phase relationships are reported with respect to a posterior reference leg and anterior observed leg (e.g., reference leg L3, observed leg L2). Full distribution is reported for *Drosophila* (gray dots); data from (Szczecinski et al. 2018); running mean for *Drosophila* is shown as a solid black line. Mean values are reported in other species; data from papers as cited in the key. For studies in which distributions were made available, only means from normally distributed phase offsets are reported (see Methods section). Shaded regions show expected phase offsets for characterized ICPs; note that labels correspond to ICPs named in hexapodal locomotion. Wave coordination shows $\phi_{I}=\frac{1}{6}$ in hexapods; more generally, metachronal waves in animals with *n* legs can show far lower phase offsets, up to a lower limit of $\phi_{I}\geq \frac{1}{n}$. Ipsilateral offsets close to 0 are observed, for example, in slow-moving millipedes (Manton 1954). Tetrapod-like coordination in hexapods shows characteristic phase offsets of $\phi_{I}=\frac{2}{6}=\frac{1}{3}$. At fast speeds, many arthropods use a stepping pattern in which two consecutive sets of legs lift-off in sequence ($\phi_{I}=\frac{1}{2}$); this corresponds to, for example, tripod coordination in hexapods ($\phi_{I}=\frac{3}{6}$). Fast-running maxillopeds, including several species of centipedes, can utilize wave coordination to achieve speeds far higher than possible in hexapods. Note that all characterized patterns across speeds and body plans maintain anti-phase contralateral coupling, $\phi_{C}=\frac{1}{2}$.

Studies in walking *Drosophila* show ICPs that merge into a speed-dependent continuum. Slow-walking flies move with a pentapodal wave coordination, in which only 1 leg is in the swing phase (lifted off the ground) at a time. At higher speeds, flies adopt a tetrapodal stepping pattern, in which two legs are in swing simultaneously. At the fastest speeds, flies almost exclusively utilize tripod coordination, in which two pairs of three legs swing in sequence (Fig. 3). The large variation observed in *Drosophila* ICPs precludes the existence of sharp switches in coordination at characteristic speeds (Fig. 4); instead, flies often can make use of multiple ICPs at the same walking speed (Mendes et al. 2013; Wosnitza et al. 2013; DeAngelis et al. 2019).

Investigations into the existence of such a continuum in invertebrate walking are crucial for understanding the underlying control strategies used by these animals, and for any attempt to compare and contrast these strategies with those well-characterized in vertebrates. For example, the generation of a multi-attractor system (as would be implied by the existence of discrete stepping patterns with discontinuous transitions between them) requires a vastly different structure than that of a single-attractor system, in which prescribed ICPs are in fact cases along a continuum.

How is such a continuum of coordination patterns generated? Based on data gathered from both slow- and fast-walking insects (the stick insect *Carausius morosus*: (Wendler 1964) and *P. americana*: (Hughes 1957)), Wilson (1966) put forward a set of simple observations hypothesized to replicate all observed insect stepping patterns, as well as the transitions between them.

1. The swing phase is initiated in a posterior to anterior wave along each ipsilateral side.
2. Contralateral leg pairs move in anti-phase.
3. The duration of swing phase within each stride is constant and independent of walking speed.
4. Stride frequency increases with speed and is modulated by changing stance duration.

These “rules” support early observations by Manton (1950, 1954, 1972), whose extensive investigations into panarthropod walking similarly noted many common features among species ). Recent work by DeAngelis et al. characterized the structure of variability in fly walking across speeds and showed that animals can seamlessly transition between canonical ICPs by modifying stance duration (Fig. 3), in support of Wilson’s final observation. Varying this single parameter also suffice to describe extensions of the ICP continuum beyond tripod coordination in fast-running hexapod species. In these cases (seen, e.g., in cockroaches, beetles, and ants (Hughes 1952; Full and Tu 1991; Wahl et al. 2015)), bipod and monopod stepping patterns are generated via the continuously increasing overlap of the swing phases of two sets of tripod legs (DeAngelis et al. 2019).

Detailed behavioral studies in *C. morosus* also largely agreed with Wilson’s observations and proposed a small set of locally distributed coordination rules (“Cruse’s rules”) which describe how a leg affects the likelihood of the initiation of a swing event in an anterior or contralateral neighboring leg (Cruse 1990; Dürr et al. 2004). *Rule 1* states that a leg’s stance-to-swing transition is suppressed while its neighbor is in swing, while *Rule 2* states that the likelihood of lift-off increases once the neighboring leg touches down. While not explicitly tested in *Drosophila*, it is quite likely that Cruse’s rules would suffice to generate the spectrum of walking behavior characterized in flies (Mendes et al. 2013; Wosnitza et al. 2013).

Our recent work on the tardigrade *H. exemplaris* confirmed the existence of these rules in the stepping patterns of freely walking tardigrades during forward walking on agarose gel substrate (Nirody et al. 2021). Stepping patterns in organisms with (many) more than six legs also generally follow the above observations and “rules” without undergoing the exact transitions shown in Fig. 3. For example, similar locomotor control circuits in myriapods manifest as a metachronal wave coordination across all walking speeds, in which the phase offset between ipsilateral legs increases with increasing speed (Manton 1950, 1952b, 1954; Kuroda et al. 2018; Yasui et al. 2019). In these systems, reducing stance duration increases the frequency of the traveling wave of swing initiations and a decrease in the number of legs involved in each cycle $n_{\text{cycle}}$ (i.e., the “wavelength”). This results in an increase in the ipsilateral phase offset $\phi_{I}=\frac{1}{n_{\text{cycle}}}$ as walking speeds increase. This may further support the hypothesis that intrinsic coordination patterns in forward walking are shared not only among insects but across panarthropod taxa.

### Galloping and other such surprises

In a set of organisms as diverse in morphology, habitat, and behavior as panarthropods, extraordinary cases will arise that deviate from any devised set of “rules.” This is inevitable regardless of how “fundamental” or general these rules purport to be. Understanding *how* and *why* certain examples shift away from seemingly “universal” traits often serves not only to characterize these exceptions but to further illuminate and refine the rule. For example, several stepping patterns observed in walking *Drosophila—*for example, an ICP in which contralateral fore- and hind-limbs swing together while each mid-limb swings alone—initially seemed distinct from previously described canonical ICPs. However, this “non-canonical” pattern, among several others, cleanly fits within the context of a continuum of limb coordination (Wilson 1966; DeAngelis et al. 2019). Similarly, the same coordination rules are active in species with more than 6 legs (Manton 1950, 1952b, 1954; Kuroda et al. 2018; Yasui et al. 2019), as well as in insects that walk less than 6 legs. For example, in case of leg loss (Grabowska et al. 2012; Wosnitza et al. 2013) or in organisms like mantids which often hold up their forelimbs and walk with the other two pairs (Wilson 1966), the same coordination rules apply simply with the missing legs omitted. However, the unique morphology of certain groups may drive a separation from this spectrum: the hydraulic extensor system in spider legs, for example, is believed to underlie several kinematic differences between Arachnida and other arthropod groups (for more details see, e.g., Weihmann et al. 2012, Booster et al. 2015; Hirt et al. 2017; Weihmann 2020; Boehm et al. 2021).

Indeed, examples that fall beyond this spectrum can be observed in several panarthropod groups. For example, several species of arthropods (cockroaches: (Weihmann et al. 2017), mites: (Weihmann et al. 2015), spiders: (Weihmann 2013)) switch to metachronal coordination at the highest observed running speeds. This switch results in a discontinuous switch in phase relationship between leg pairs and is hypothesized to be advantageous for locomotion on slippery surfaces (Weihmann et al. 2017). In the alternating tripod, lateral ground reaction forces (GRFs) generated by the front and middle legs within each tripod brace against each other (Dickinson et al. 2000). This may contribute to energy recovery during a stride, as well as to dynamic stability by controlling the lateral dynamics of the COM (Schmitt et al. 2002; Weihmann et al. 2017). However, these benefits are largely absent when moving on slippery or granular substrates; here, these lateral forces can risk slipping (Li et al. 2009). The observed metachronal pattern, however, constitutes a desynchronization of the legs within each alternating tripod set (Weihmann et al. 2017), removing the detrimental effects of double stance on flowing or slippery media (Li et al. 2009). Additionally, vertical forces and impulses from leg impacts are high during tripod stepping patterns, which constitute only two steps (of three legs each) per stride; these vertical GRFs are reduced and more evenly distributed among limbs in metachronal patterns (Li et al. 2009, 2013). Furthermore, lifting the requirement for three legs to step simultaneously increases the temporal overlap between the stance periods of consecutive sets of legs, allowing for the duty factor to decrease without an aerial phase (Weihmann et al. 2017). Maintaining permanent ground contact may have additional advantages on slippery substrates, for example, because it allows for uninterrupted proprioceptive input on the animal’s position with respect to the ground (Sponberg and Full 2008).

One of the most prevalent features noted in ICPs across species is that swing initiations occur in a posterior to anterior wave on each ipsilateral side ($\phi_{I}<0.5$); Wilson’s first observation noted that this pattern manifests across all walking speeds in several insect species (Wilson 1966; Cruse 1990). In fact, this “rule” holds true across panarthropods with very few exceptions, the majority of which are within the class Chilopoda (centipedes). Myriapods all progress using “locomotory waves”; millipedes (class Diplopoda) and two of the five orders of centipedes display the expected posterior-to-anterior pattern (Manton 1954; Kuroda et al. 2018). However, the other three centipede orders (Craterostigmorpha, Scolopendromorpha, Geophilomorpha) exhibit *retrograde* waves: swing initiations that occur in an anterior-to-posterior manner ($\phi_{I}>0.5$) (Manton 1952; Kuroda et al. 2018). Molecular phylogenies of Myriapoda indicate that retrograde waves may be a derived feature (Miyazawa et al. 2015; Fernández et al. 2018); further ecological, functional, and anatomical studies into these centipede orders will be needed to understand both the selective factors and the underlying neural basis for the determination of wave direction.

Contralateral coupling is generally more variable than coupling between ipsilateral leg pairs, both within a single species and among different panarthropod species. In particular, several species across diverse panarthropod taxa exhibit in-phase contralateral coordination, rather than anti-phase as in Wilson’s second observation (Wilson 1966). Aquatic species (e.g., krill (Zhang et al. 2014) and water striders (Bowdan 1978)) display in-phase contralateral strokes while moving under or on the surface of the water, a coordination pattern believed to be highly optimized for aquatic locomotion (Zhang et al. 2014; Takagi 2015). Many species of millipedes similarly show a preference for in-phase contralateral coupling, a pattern that has been measured to provide increased pushing force during burrowing (Manton 1954, 1958). Synchronous contralateral coordination in terrestrial arthropods has rarely been observed other than in transient situations, for example, when traversing three-dimensional terrain or for the first few steps when walking is first initiated (Pearson and Franklin 1984). However, recent observations in three species of flightless dung beetles in the genus *Pachysoma* noted a “galloping” coordination pattern in which contralateral leg pairs step in phase with each other (Smolka et al. 2013). Interestingly, galloping species of *Pachysoma* are not faster than their tripodal siblings, suggesting that there is no speed advantage to this stepping pattern (Smolka et al. 2013). In support of the hypothesis that in-phase contralateral swings may provide some advantage on shifting substrates like the sands desert-dwelling *Pachysoma* must traverse, we observed sustained “gallops” in tardigrades walking on substrates of reduced stiffness (∼10 kPa) (Nirody et al. 2021).

### A simple framework for the panarthropod ICP continuum

**Fig. 5.**
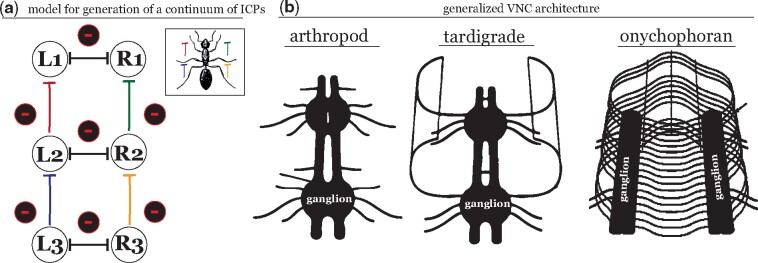
A simple model for the generation of ICPs based on VNC architecture. (**A**) Detailed characterization of *Drosophila* coordination patterns across walking speeds in (DeAngelis et al. 2019) suggests that a simple control circuit may be sufficient to generate all observed ICPs in walking *Drosophila*. The proposed circuit comprises mutual inhibitory connections between contralateral leg pairs and a posterior-to-anterior inhibition on each ipsilateral side; inhibitory connections are denoted by capped vertical lines with associated (−) signs. These connections are postulated to be found in the thoracic ganglia of the *Drosophila* VNC. (**B**) VNC structure in arthropods and tardigrades consists of a series of segmented ganglia, each of which corresponds to a single leg pair. Onychophorans have two laterally located ganglia connected by median commissures at each leg pair. The conserved general topology of VNC architecture across Panarthropoda lends support to the possibility that the functional similarities in stepping patterns observed in these diverse taxa may originate from a shared underlying control strategy. (**B**) Modified from (Yang et al. 2016).

A large variety of theoretical and computational models have been developed over the years to describe stepping patterns in hexapod locomotion (Cruse 1990; Ijspeert 2008; Aminzare et al. 2018; Szczecinski et al. 2018; Schilling and Cruse 2020). Based on a comprehensive analysis of the variability in *Drosophila* leg coordination across walking speeds, DeAngelis et al. propose that a single continuum can describe all observed patterns in fly walking (DeAngelis et al. 2019). As previously mentioned, such a continuum, which does not need to account for multiple discrete coordination patterns, allows for the possibility of a simpler control circuit underlying forward walking in *Drosophila*. This simple model suggests the existence of mutual inhibitory coupling between contralateral neuropil and posterior-to-anterior inhibitory coupling between ipsilateral neuropil of the VNC in *Drosophila* (Fig. 5a). Excitingly, DeAngelis et al. also show that varying a single parameter, stance duration, can replicate fundamental components of the observed spectrum of ICPs without any speed-dependent modulation of ipsilateral and contralateral coupling (Fig. 3).

The ICPs observed in the *Drosophila* continuum closely mirror features of those in a range of insects and other arthropods, as well as those recently characterized in tardigrades (Fig. 4a). Panarthropod groups display notable similarities in VNC architecture (Fig. 5b). This may intriguingly support the existence of a shared underlying locomotor control circuit in Panarthropoda, which has been modified along with certain clades due to specific pressures on organismal performance (Yang et al. 2016). The VNC in onychophorans shows several differences from that of tardigrades and arthropods, containing ladder-like lateral ganglia connected by interpedal median commissures (Fig. 5b). However, the topology of this structure is not significantly different from the segmented hemiganglia of tardigrades and arthropods and does not rule out the existence of a shared control circuit between onychophorans and the other panarthropod taxa (Yang et al. 2016). Previous observations of onycophoran locomotion determined that average ipsilateral phase offsets are consistent with those of other panarthropods (Fig. 4a); coupling between contralateral leg pairs is irregular at low speeds but converges to anti-phase contralateral coupling at high speeds (Manton 1950, 1952a; Oliveira et al. 2019). More detailed analyses in velvet worm species are needed to reveal how, if at all, morphological differences between the VNC of Onychophora and Tardigrada + Arthropoda affect inter-leg coordination.

Molecular studies have found that the compact tardigrade body plan evolved from a loss of a large body region corresponding to the entire thorax and part of the abdomen in arthropods. This indicates that the tardigrades’ legged segments are homologous only with the head region of other panarthropods (Smith et al. 2016). These results support the hypothesis that the diversity of head appendages in arthropods and onychophorans evolved from legs (Eriksson et al. 2010; Angelini et al. 2012). While this does not necessarily preclude the idea that a common circuit underlies forward walking in panarthropods, the alternative hypothesis is that similarities in tardigrade and arthropod coordination patterns have independently evolved. This parallel convergence onto similar inter-leg coordination strategies by these two groups is intriguing, given their remarkable disparities in size and skeletal structure and may provide significant insight into general design principles for efficient and robust control of multi-legged locomotion. A more definitive distinction between these scenarios will require deeper functional studies combined with molecular and phylogenetic analyses.

In accordance with observations made by Wilson decades prior (Wilson 1966), we note several key features of an “idealized” ICP spectrum. First, as noted previously, only stance duration is varied with walking speed; the duration of the swing phase is largely speed-independent. Second, ipsilateral swings of adjacent legs do not overlap and occur in a posterior-to-anterior wave. In an animal with *n* legs, this results in the phase offset between ipsilateral legs increasing from $\phi_{I}\geq \frac{1}{n}$ at the lowest walking speeds up until a maximum offset of $\phi_{I}=0.5$ at the fastest speeds ($\phi_{I}>0.5$ corresponds to a retrograde wave of swing initiations that travels posteriorly). In the case of hexapods, this corresponds to a speed-dependent continuum varying smoothly from $\phi_{I}=\frac{1}{6}$ in wave coordination to $\phi_{I}=\frac{1}{3}$ in tetrapod to $\phi_{I}=\frac{1}{2}$ in tripod coordination (Fig. 3). Finally, contralateral leg pairs show a preference for anti-phase coordination $\phi_{C}=\frac{1}{2}$ across all walking speeds (Fig. 4b).

Of course, measurements in freely behaving animals rarely adhere to any semblance of “ideal” relationships. One such deviation arises from the stipulation that contralateral legs prefer anti-phase coordination. The “canonical” tetrapod comprises a sequence of swing initiations by three sets of two legs; this results in a contralateral offset of $\phi_{C}=\frac{1}{3}$ (or $\phi_{C}=\frac{2}{3}$ for the mirror-image tetrapod) at lower speeds. DeAngelis et al. (2019) report a cross-body offset in step timing such that limbs that are meant to swing simultaneously are actually slightly offset in time, resulting in an anti-phase contralateral phase relationship (Fig. 3). Nearly all surveyed arthropod species similarly showed, on average, anti-phase contralateral phasing (Fig. 4b; although several exceptions are noted in the section above). Of course, this may result from a bimodal distribution with peaks at $\frac{1}{3}$ and $\frac{2}{3}$ corresponding to the 2 mirror-image tetrapods. However, all studies in which complete data was made available reported contralateral phase-offset distributions with a single peak centered around $\phi_{C}=\frac{1}{2}$. Further investigation into this relationship across taxa will be needed to confirm the generalizability of this simple model.

Measured inter-leg relationships also show high variability (Fig. 4). Interestingly, all pairwise inter-leg relationships show higher variability at low speeds than during fast walking. A possible explanation for this pattern is purely physical: when limbs have asymmetric duty cycles, they cover different fractions of a cycle per unit time when in swing versus stance. As such, slow walking, which has longer stance phases and approximately the same swing duration as fast walking, will show greater variance in relative phasing (Couzin-Fuchs et al. 2015; DeAngelis et al. 2019).

An alternative is that inter-limb coupling is more affected by sensory information at low speeds than at high speeds, and thus is more variable (Schilling and Cruse 2020). This explanation is consistent with observations of higher variability in limb coordination patterns in slow-walking insects when compared with fast runners (Delcomyn 1991; Sponberg and Full 2008; Bidaye et al. 2018). We note that this option does not necessarily require a speed-dependent modulation of inter-limb coupling strength: there is a fundamental timescale related to the propagation of sensory information, which is too slow to drive behavior at speeds higher than approximately 5 strides per second (corresponding to a stepping period of ∼200 ms) in *P. americana* (Delcomyn 1991; Schilling and Cruse 2020); this limit may be higher in *Drosophila* due to its relatively smaller size.

Contralateral coordination is generally weaker than ipsilateral coordination across all surveyed species (Nirody et al. 2021). Studies in a range of organisms have shown flexibility in the coupling between contralateral leg pairs within single individuals in response to external stimuli; for example, we characterized a transition from anti-phase to in-phase contralateral coupling in *H. exemplaris* with changes in substrate stiffness with no shift observed in ipsilateral phase offsets (Nirody et al. 2021). Furthermore, the relative weakness of coupling between contralateral leg pairs in comparison with ipsilateral leg pair coupling is consistent with the hypothesis that the underlying controller proposed based on *Drosophila* is shared across panarthropods. Contralateral phasing is quite variable across taxa, ranging from in-phase in swimming Crustacea (Zhang et al. 2014) to anti-phase in running insects (Full and Tu 1991; Merienne et al. 2021) and arachnids (Spagna et al. 2011; Weihmann et al. 2015). However, ipsilateral phase relationships are consistent across nearly all characterized species (with few exceptions; see, e.g., (Manton 1952b; Kuroda et al. 2018)).

To further substantiate how the walking system characterized in *Drosophila* compares with that in other insects, and for panarthropods in general, it will be necessary to undertake deeper comparative investigations. We note that this review focused on leg kinematics during forward walking on flat surfaces; far less comparative data was available for body and center-of-mass dynamics, as well as for more complex behavior such as turning, obstacle traversal, backward walking, and loaded locomotion. Intriguingly, there is evidence that turning in *Drosophila* requires only a small modification of the hypothesized forward walking circuit (DeAngelis et al. 2019); this remains to be tested in other animals. As tools for automating collection and analysis of large behavioral datasets become more commonplace (Mendes et al. 2013; DeAngelis et al. 2019; Pereira et al. 2019), the goal of intensive and comprehensive characterization of walking across panarthropod taxa comes within reach. However, crucial to the success of such studies is the accessibility of raw movement data in a wide range of species; we hope that this work sheds light on the importance of these analyses.

## Notes

From the symposium “Physical mechanisms of behavior” presented at the virtual annual meeting of the Society for Integrative and Comparative Biology, January 3–7, 2021.
